# Tuft Cells Increase Following Ovine Intestinal Parasite Infections and Define Evolutionarily Conserved and Divergent Responses

**DOI:** 10.3389/fimmu.2021.781108

**Published:** 2021-11-22

**Authors:** Katie A. Hildersley, Tom N. McNeilly, Victoria Gillan, Thomas D. Otto, Stephan Löser, François Gerbe, Philippe Jay, Rick M. Maizels, Eileen Devaney, Collette Britton

**Affiliations:** ^1^ Institute of Biodiversity, Animal Health and Comparative Medicine, University of Glasgow, Glasgow, United Kingdom; ^2^ Disease Control Department, Moredun Research Institute, Penicuik, United Kingdom; ^3^ Wellcome Centre for Integrative Parasitology, Institute of Infection, Immunity and Inflammation, University of Glasgow, Glasgow, United Kingdom; ^4^ Institut de Genomique Fonctionnelle (IGF), University of Montpellier, CNRS, INSERM, Montpellier, France

**Keywords:** Tuft cell, parasitic nematode, immunity, gastrointestinal tract, single cell RNA sequencing, RNAscope, immunohistochemistry, G protein-coupled receptor

## Abstract

Helminth parasite infections of humans and livestock are a global health and economic problem. Resistance of helminths to current drug treatment is an increasing problem and alternative control approaches, including vaccines, are needed. Effective vaccine design requires knowledge of host immune mechanisms and how these are stimulated. Mouse models of helminth infection indicate that tuft cells, an unusual type of epithelial cell, may ‘sense’ infection in the small intestine and trigger a type 2 immune response. Currently nothing is known of tuft cells in immunity in other host species and in other compartments of the gastrointestinal (GI) tract. Here we address this gap and use immunohistochemistry and single cell RNA-sequencing to detail the presence and gene expression profile of tuft cells in sheep following nematode infections. We identify and characterize tuft cells in the ovine abomasum (true stomach of ruminants) and show that they increase significantly in number following infection with the globally important nematodes *Teladorsagia circumcincta* and *Haemonchus contortus*. Ovine abomasal tuft cells show enriched expression of tuft cell markers *POU2F3, GFI1B, TRPM5* and genes involved in signaling and inflammatory pathways. However succinate receptor *SUCNR1* and free fatty acid receptor *FFAR3*, proposed as ‘sensing’ receptors in murine tuft cells, are not expressed, and instead ovine tuft cells are enriched for taste receptor *TAS2R16* and mechanosensory receptor *ADGRG6.* We also identify tuft cell sub-clusters at potentially different stages of maturation, suggesting a dynamic process not apparent from mouse models of infection. Our findings reveal a tuft cell response to economically important parasite infections and show that while tuft cell effector functions have been retained during mammalian evolution, receptor specificity has diverged. Our data advance knowledge of host-parasite interactions in the GI mucosa and identify receptors that may potentiate type 2 immunity for optimized control of parasitic nematodes.

## Introduction

Parasitic nematodes induce long lasting chronic infections in their hosts, driven by expansion of Th2 and regulatory T cells (reviewed in reference 1). Most studies of parasite infection have concentrated on mouse models; similar responses occur in livestock animals, but are less well understood (2, 3). Recent studies in mice infected with the gastrointestinal (GI) parasitic nematodes *Nippostrongylus brasiliensis* and *Heligmosomoides polygyrus* demonstrated the importance of tuft cells, a rare and unusual type of intestinal epithelial cell, in initiating the type-2 response in the small intestine (SI) (4–6). Tuft cells exclusively secrete the alarmin IL-25 within the epithelium, leading to activation of type-2 innate lymphoid cells (ILC2), which secrete IL-4 and IL-13 required for Th2 expansion (7). IL-4 and IL-13 then act in a feed-forward loop to induce tuft and goblet cell differentiation, leading to further IL-25 release (4–6). Increased goblet cell mucus production and gut contractility result in helminth expulsion from the GI tract, referred to as the “weep and sweep” response (8). In mice, tuft cells are proposed to act as sentinels of GI mucosal immunity, sensing the presence of parasites and triggering the type 2 response (4–6, 9–11). While the tuft cell-ILC2 axis was identified in murine SI, nothing is known of tuft cells in immunity in other host species and in other regions of the GI tract. To address this gap we have examined the presence and characteristics of tuft cells following ovine nematode infections.

Tuft cells were first characterized by their unusual morphology, including an apical cluster or “tuft” of microvilli projecting into the gut lumen (12), subsequently shown to be linked to the endoplasmic reticulum by a tubulo-vesicular network (13). Tuft cells have also been characterized by expression of specific marker proteins including transcription factors POU2F3 and GFI1b, Doublecortin-like kinase DCLK-1, associated with tuft cell microtubules, and receptor potential cation channel TRPM5, a component of the tuft cell chemosensory machinery (4, 14–16). Brush or border cells with a tuft-like extension were previously reported in ruminant epithelia, but their specific roles are unknown (17). Livestock ruminants, particularly young animals, are highly susceptible to GI nematode infections, which negatively impact health and productivity, costing the livestock industry approximately GBP 10 billion per annum globally in productivity losses and treatments (18). Anthelmintic resistance is a serious threat to the control of GI nematodes (19), including the prevalent ovine abomasal dwelling nematodes *Teladorsagia circumcincta* and *Haemonchus contortu*s, related to human hookworms. Alternative control strategies are needed, with efforts increasing in vaccine development (2). It is therefore important to better understand mechanisms of immunity in the GI tract and how these may be potentiated for optimal parasite control.

Here we describe for the first time the presence and expansion of tuft cells following GI nematode infection in the sheep abomasum, the true stomach of ruminants, which represents the simple monogastric stomach in structure and function. We use single cell RNA sequencing (scRNA-seq), to detail tuft cell gene expression, with the long term aim of defining molecules and mechanisms that elicit tuft cell expansion. We demonstrate the expression of genes required for leukotriene and prostaglandin synthesis and G-protein signaling, establishing that mediators of tuft cell function are conserved across species and GI tissues. In contrast, receptors that can sense pathogens are divergent. We also provide a cell atlas of gene expression in the ovine abomasal mucosa that greatly extends current ovine genomic and functional annotation (20). Our findings advance knowledge of GI immunity to parasites, and the receptors identified here have potential in development of prophylactic treatment to control these globally important infections.

## Materials and Methods

### Tissue Samples and Ethics Statement

All animal procedures were performed at Moredun Research Institute (MRI), UK under license as required by the UK Animals (Scientific Procedures) Act 1986, with ethical approval from MRI Animal Experiments Committee. Animals were raised at MRI under conditions designed to exclude accidental infection with helminth parasites and were helminth-naïve.

Abomasal tissue for IHC was collected over a time course of *T. circumcincta* infection with six animals per group, matched for weight and sex (Experiment 1, **Supplementary Table 1**). For IHC after *H. contortus* infection, abomasal tissue was collected at day 55 p.i. with 10,000 L3 (Experiment 2, **Supplementary Table 1**). Abomasal tissue for scRNA-seq (Experiment 3, **Supplementary Table 1**) was collected at day 21 p.i. from two lambs infected with 50,000 *T. circumcincta* L3. Small intestine tissue samples were collected from three lambs infected with 7,000 *H. contortus* L3 and tissue collected at day 56 post-infection. Sections of murine SI and ovine brain tissue were obtained from studies at MRI and were included in IHC to test specificity and species cross-reactivity of the DCLK-1 antibody.

### Immunohistochemistry (IHC) and Mucin Staining

Antibodies are detailed in **Supplementary Table 2**. Abomasal tissue sections were fixed in 4% paraformaldehyde for 6 hours at room temperature (RT) and embedded in paraffin wax (PFPE). Following dewaxing, antibody staining was carried out as described previously (21). For double immunofluorescence, following antigen retrieval and blocking, slides were incubated overnight at 4°C with mouse anti-GFI1b. HRP labelled anti-mouse DAKO Envision™+ Polymer (Agilent) was applied for 30 min at RT followed by incubation with Alexa Fluor^®^ 488 Tyramide substrate (Invitrogen). Slides were then incubated with anti-POU2F3 antibody, washed and incubated with goat anti-rabbit IgG (H+L) conjugated to Alexa Fluor-546^®^ (Thermo Fisher Scientific). Hoechst stain (Invitrogen) was applied for 5 min and Prolong Gold (Invitrogen) added before viewing under a Zeiss Axiovision fluorescence microscope.

Periodic Acid Schiff (PAS) Stain kit (HS462, TCS Biosciences) was used to stain mucins. Tissue sections were incubated with 0.3% hydrogen peroxide-PBST, washed, then PAS added for 5 min. Following rinsing, Feulgen Stain (Schiff) was added for 15 min, slides washed, and stain left to develop for 10 min.

### Microscopy and Imaging

An Olympus BX50 Microscope (Model U-SD0, Olympus Optical Co., Ltd.) with an Olympus DP70 camera and Olympus U-CMAD3 adapter was used with the AnalySIS program for imaging. For enumeration of POU2F3^+^ cells, five images at x40 magnification were acquired for each tissue section, ensuring no overlap and samples scored blind. All epithelial cells were counted using Image J (22). Following counting of all POU2F3^+^ cells within the epithelial cells, the percentage of POU2F3^+^ cells/all epithelial cells was calculated. The mean percentage of POU2F3^+^ cells was calculated across the five images for each sheep.

### Statistical Analysis

R version 4.0.3 using the statistical package R Core Team (https://www.R-project.org/) was used unless otherwise stated. For *T. circumcincta* time-course infection, IHC cell count data were analyzed using a Kruskal-Wallis test followed by Dunn’s multiple comparisons test to compare median values where data were not normally distributed. Two-sample t-tests were used to compare mean values for normally distributed data. Generalised Linear Mixed Models (GLMM) were used to examine effects of abomasal region and infection status on percentage of tuft cells.

### RNAscope on Ovine Abomasal Tissue to Validate scRNA-Seq Data

RNAscope 2.5 HD Duplex Detection Kit (Advanced Cell Diagnostics (ACD), Bio-Techne, UK) was used to perform *in situ* hybridization on abomasum tissue collected from three sheep; two helminth-naïve and one sheep at 30 days post-*H. contortus* infection. Due to differences in fixation time (6 h for IHC, 24 h for RNAscope) we were unable to use the tissue collected following *T. circumcincta* infection for RNAscope. We had access to *H. contortus*-infected and control (uninfected) tissue fixed for the requisite time and we made use of these samples for validation of scRNA-seq data. Probes were designed by ACD to target ovine *POU2F3* (green signal) and ovine *IL17RB*, *TAS2R16* and *DCLK-2* (fast red signal). Positive control probes were designed to target ovine beta actin (*actb*; green signal) and ovine peptidylprolyl isomerase (*ppib*; red signal). Negative control probe was designed to *DapB* from *Bacillus subtilis*. Ovine abomasum tissue was fixed in 10% formalin for 24 hours, moved to 70% ethanol, then processed and embedded into paraffin wax blocks. Sections (4 μM) were mounted onto Superfrost Plus glass slides. RNAscope was performed following manufacturer’s instructions, with optimization for ovine abomasum tissue and signal enhancement. Target retrieval was applied for 7 min, hydrogen peroxide for 10 min, followed by Protease Plus for 15 min. For *IL17RB* and *TAS2R16* probes, the Amp 5 step was reduced to 10 min to optimize the signal. Slides were counterstained with Haematoxylin and imaged using light microscopy equipment described above.

### Expression of Recombinant POU2F3 and Western Blot Analysis

Full-length *Ova-POU2F3* gene (NCBI XM_015100962.2) with C-terminal enhanced yellow fluorescent protein (eYPF) tag was obtained from Eurofins. *Ova-POU2F3* insert was ligated into *Xho*I-*Not*I digested pCI-neo vector (Promega) and plasmid DNA from the pCI-neo vector alone, pCI-neo containing *Ova-POU2F3* or pCAG-neo containing ovine transcription factor *FOXP3* gene (23) was transformed initially into Top10 cells, then into HEK293 cells. For western blot analysis, cells were lysed in NuPAGE sample buffer (Invitrogen) with 2% β-mercaptoethanol, proteins separated on a Mini-PROTEAN TGX Precast Protein Gel (Bio-Rad), then transferred to nitrocellulose membrane. Following Ponceau S staining, the membrane was cut between the 50 and 75 kDa marker proteins, blocked (5% milk powder in 0.1% PBS-Tween20, PBST), and probed overnight at 4°C with primary antibody: anti-POU2F3, 1:2500 (Sigma-Aldrich, HPA019652) or anti-actin, 1:1000 (Sigma, A-3853) before incubation for 1 hour at RT with secondary antibody: anti-mouse peroxidase, 1:20,000 (Sigma, A-2304) or anti-rabbit peroxidase, 1:10,000 (Sigma, A-0545). Following washing in PBST, membranes were re-joined and signal developed using SuperSignalTM West Pico PLUS Chemiluminescent Substrate (Thermo Scientific) and captured on x-ray film.

### Single-Cell Sample Preparation

Ovine abomasum epithelial cells were isolated from six 3 cm^2^ gut fold sections collected at post-mortem from two sheep 21-days post *T. circumcincta* infection, following 10x Genomics single cell protocol. Tissue sections were placed in ice-cold Hanks’ Balanced Salt Solution (HBSS) containing 2.5% fetal bovine serum (FBS), two sections of tissue per tube. Tissue was washed with HBSS, shaking gently to remove any stomach contents, then incubated with HBSS containing 2.5 mM EDTA (Sigma Aldrich), 1mM DTT (Sigma Aldrich) and 10 μg/ml DNase I (Roche) at 37°C for 20 min with agitation. Tubes were shaken vigorously, and the supernatant centrifuged at 330 x *g* for 5 min to pellet released cells, which were washed in HBSS, centrifuged again and re-suspended in HBSS containing 1 U/ml Dispase II (Stem Cell Technologies, 07913) and 10 μg/ml DNase I (Sigma, 10104159001) and incubated for 10 min at 37°C with agitation. FBS (250 μl) was added to each tube, which was then centrifuged. Following a final wash in HBSS, cells from the same sheep were pooled in 5 ml HBSS and strained through a 70 μm Nylon mesh sterile strainer (Fisherbrand). FBS (250 μl) was added, samples centrifuged and pellets re-suspended in 5 ml PBS. Cell count and viability assessment were performed with Trypan Blue staining.

### 10x Genomics Sample Processing, cDNA Library Preparation and Initial Analysis

The cell suspension for each sheep was processed separately using the droplet-based Chromium Controller microfluidic platform (10x Genomics) (24) with Chromium Single Cell 3’ Reagent Kit (v.3) according to the manufacturer’s protocol by University of Glasgow Polyomics facility using a total of 20,000 input cells in a volume of 46 μl. Briefly, cells were partitioned into Gel Beads in Emulsion, followed by lysis, and extracted RNA subject to barcoded reverse transcription. cDNA was amplified (12 cycles), libraries generated and sequenced on an Illumina NextSeq 500 system to a depth of 50,000 read pairs per cell. Sequences were mapped to the genomes for ovine (*Ovis aries* Oar_v3.1; https://www.ensembl.org/Ovis_aries/Info/Index) and bovine (*Bos taurus* ARS-UCD1.2; https://www.ensembl.org/Bos_taurus/Info/Index) separately and the respective gene counts matrices generated using Cell Ranger software (version 3.1.0). As only approximately 50% of transcripts had annotated 3’UTR, we extended the 3’UTR end of each transcript by 1500 bp.

### Single Cell RNA-Seq Data Analysis Using Seurat

Seurat package, version 3.1 (25, 26) was used with R version 4.0.3 (https://www.r-project.org). Other packages used for analysis were ggplot2, car, ggpubr (https://CRAN.R-project.org/package=ggpubr), dplyr (https://CRAN.R-project.org/package=dplyr) and cowplot (https://CRAN.R-project.org/package=cowplot).

Following the guidance of the Seurat Vignette (https://satijalab.org/seurat/v3.1/pbmc3k_tutorial.html), each sample was pre-processed, subject to QC and filters applied, based on the individual sample QC metrics. For sample 1, cells with <300 and >6500 genes were removed, to mitigate including potential doublets as well as cells with a mitochondrial gene percentage of >20%. For sample 2, cells with <300 and >5750 genes were excluded, and cells with mitochondrial gene percentage of >20%. Sample data were then transformed and normalized with the “Log Normalise” function. After processing, samples 1 and 2 contributed 7,963 and 8,928 cells, respectively, and >17,000 genes for downstream analysis. The “Find Integration Anchors” followed by the “Integrate Data” Seurat functions were used to combine the sample data. The number of significant principal components (PC = 35) was selected based on Elbow plots and applied when combining the data. Default Seurat methods (Findclusters) with resolution parameter 0.1 and UMAP were used to visualize the cells and clustering. The “Find Markers” function, with default parameters (log fold change (FC) >0.25), generated gene expression lists for each cluster based on differential expression, calculated by a non-parametric Wilcoxon rank sum test (26). Based on marker genes expressed by at least 25% of cells in the cluster, different cell populations were annotated. Use of the “RNA assay” was specified when generating plots of specific genes of interest.

### Tuft Cell Sub-Cluster Trajectory Inference and Pseudotime Analysis

To further analyze ovine tuft cells, we repeated the clustering step on these cells (PCA dimension of 15 and cluster resolution of 0.5). For trajectory inference, tuft cells were plotted using PHATE maps (27) (using the same genes as for PCA) and trajectories identified using slingshot (28), with sub-cluster 1 defined as the putative starting point. Genes with expression patterns associated with progression of the trajectory were identified using generalized additive models with tradeSeq package v1.3.18104 default parameters. Differential expression analysis was carried out using tradeSeq association Test function with default parameters, and significant genes (log FC > 0.25) clustered using tradeSeq clusterExpressionPattern.

## Results

### Identification of Ovine Abomasal Tuft Cells by Immunohistochemistry

To determine if cells characteristic of tuft cells are present in the ovine abomasal epithelium, antibodies to human POU2F3, GFI1b, TRPM5 and DCLK-1 (15) (**Supplementary Table 2**) were tested by immunohistochemistry (IHC). Analysis of ovine genome data (https://www.ensembl.org/Ovis_aries/Info/Annotation) identified sufficient conservation (**Supplementary Table 3**) to suggest antibody cross-reactivity.

**Figure 1:**
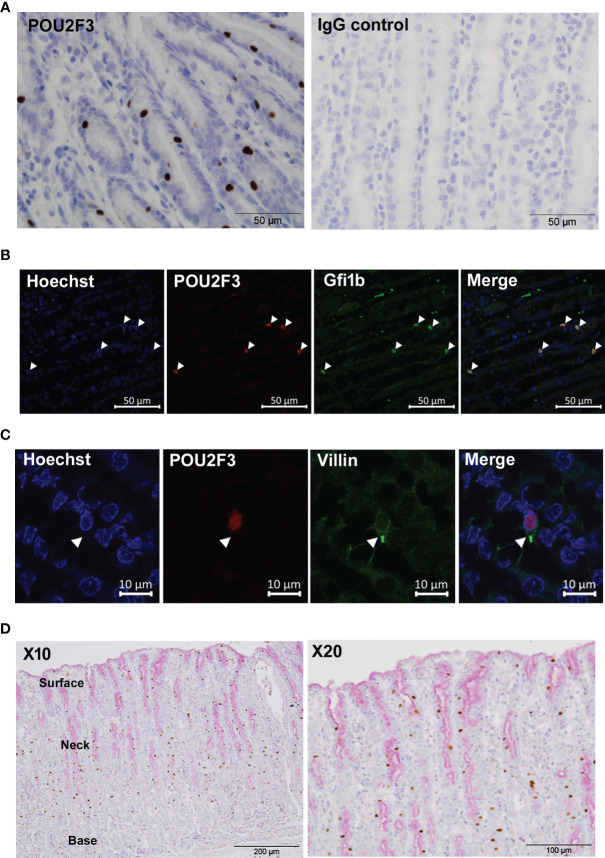
Identification of tuft cells in ovine abomasal epithelium by immunohistochemistry. **(A)** Detection of tuft cells using antibody to human POU2F3 (brown) in ovine abomasal epithelium sections at day 21 post-infection with *Teladorsagia circumcinta* (left hand panel). Surrounding epithelial cells are counterstained with hematoxylin blue. IgG isotype control antibody showed no labelling (right hand panel). **(B)** Co-localization of antibodies to POU2F3 and GFI1b in ovine tuft cell nucleus. Arrowheads indicate nuclear localization of each antibody individually and in the merged image. Nuclei were stained with Hoechst. **(C)** Detection of apical tuft on POU2F3^+^ cells (red) with antibody to villin (green). Arrowhead indicates tuft-like structure on double-labelled cell. Nuclei were stained with Hoechst. **(D)** POU2F3^+^ cells are distinct from mucin-secreting cells. Co-labelling with POU2F3 antibody (brown) and mucin stain Periodic Acid-Schiff (pink), which localizes to the surface mucus layer and to surface and neck mucous cells, predominantly at the apical (upper) edge of the abomasal epithelium. Areas of surface and neck mucous cells and the base of the gastric glands are annotated.

We first examined samples at 21 days post-infection (p.i.) with *T. circumcincta*, a time-point at which adult parasites are present in the abomasum lumen. Clear labelling of POU2F3^+^ epithelial cells, consistent with the distribution of tuft cells in mice, was observed (**Figure 1A**). Western blot of transfected HEK cells expressing ovine POU2F3 confirmed specificity of the anti-POU2F3 antibody (**Supplementary Figure 1**). Double-immunostaining with antibodies to tuft cell transcription factors POU2F3 and GFI1b showed co-localization to the cell nucleus, confirmed by Hoescht co-staining (**Figure 1B**), providing further evidence that POU2F3 positive cells were indeed ovine tuft cells. Murine tuft cells are characterized by an apical tuft, and this structure could be identified on ovine tuft cells using antibody to villin (**Figure 1C**). POU2F3^+^ cells were evenly distributed throughout the epithelial layer from the base of the gastric glands to the apical surface. This contrasted with the labelling observed following staining of mucous cells (goblet-like cells of the abomasum/stomach) and extracellular mucus with Periodic Acid-Schiff (PAS), which concentrated towards the apical surface of the epithelium where surface/neck mucous cells are located, and did not co-localize to POU2F3^+^ cells (**Figure 1D**).

Using IHC, specific staining was not observed in ovine abomasal tissue with antibodies to DCLK-1 or TRPM5, in contrast to murine SI tuft cells (4–6), suggesting possible differences in tuft cells in mouse and sheep, or between gastric and SI tuft cells. Importantly, we established that the antibodies cross-react with the putative ovine homologues: specific labelling was observed with DCLK-1 antibody in ovine hippocampus tissue (**Supplementary Figure 2A**), consistent with expression of DCLK-1 in neurons (29). DCLK-1 antibody also showed reactivity with putative tuft cells in murine SI tissue, as expected (**Supplementary Figure 2C**), but specific antibody binding was not observed in ovine abomasum, duodenum or jejunum (**Supplementary Figures 2D–F**). TRPM5 antibody localized to tuft cells in the ovine duodenum and jejunum, but not in the abomasum (**Supplementary Figures 2A–I**). These results suggest that DCLK-1 is not expressed in ovine tuft cells, and that in sheep, TRPM5 may be present in intestinal but not gastric tuft cells.

### The Frequency of Ovine Tuft Cells Increases Following GI Nematode Infections

**Figure 2:**
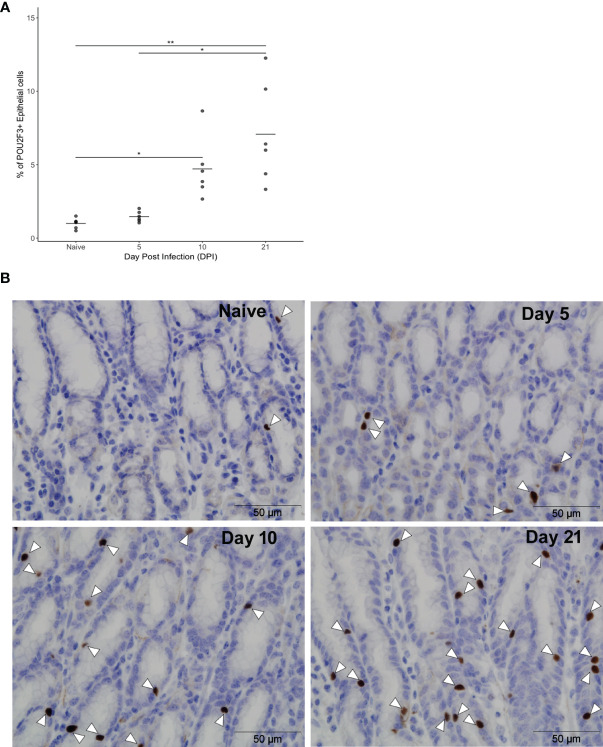
Frequency of ovine abomasal tuft cells increases following GI nematode infection. **(A)** Percentages of POU2F3^+^ cells in total epithelium in naïve sheep and at days 5, 10 and 21 following *Teladorsagia circumcincta* infection. The percentage of POU2F3^+^ cells from five areas per tissue section was calculated from each of six animals per group as detailed in **Supplementary Table 4**. Tuft cells represented 0.9% ± 0.3 (mean ± standard deviation), 1.5% ± 0.4, 4.7% ± 2.1 and 7.1% ± 3.4 of epithelial cells in the abomasum of naïve animals and at days 5, 10 and 21 p.i., respectively. * indicates P ≤ 0.05; ** indicates P ≤ 0.01. P values were calculated using a Dunn’s Multiple comparison test. Horizontal lines indicate mean values. **(B)** Representative images of POU2F3^+^ cells (brown) in the ovine abomasum epithelium from naïve sheep and at days 5, 10 and 21 after *T. circumcincta* infection. White arrowheads indicate POU2F3^+^ cells.

The kinetics of the ovine tuft cell response to nematode infection was investigated using anti-POU2F3 antibody on abomasal tissue from *T. circumcincta*-infected sheep at days 5, 10 and 21 p.i. (Experiment 1, **Supplementary Table 1**). These time points capture key events in *T. circumcincta* development: at day 5 L4 stage larvae are present within the gastric glands, at day 10 immature adults are beginning to emerge from the glands, and at day 21 mature adults are present within the abomasal lumen (30). A significant increase in POU2F3^+^ cells was first observed at day 10 p.i. relative to helminth-naive counterparts, with a 5.2-fold increase at day 10 and 7.9-fold increase at day 21 compared to naïve animals (P < 0.05 and P < 0.01 for days 10 and 21 p.i., respectively) (**Figures 2A, B**, and **Supplementary Table 4**). This is similar to data from mouse SI where an 8.5-fold increase in tuft cells was observed following *N. brasiliensis* infection (4).

We also examined, albeit at a later time point, tuft cells in sheep infected with *H. contortus* (**Supplementary Table 1**), a highly pathogenic blood-feeding abomasal nematode related to human hookworms. Significantly more POU2F3^+^ cells were present in the abomasal epithelium at 55 days p.i. compared to uninfected sheep **(** **Supplementary Figure 3**). This increase was lower than observed at day 21 of *T. circumcincta* infection which may reflect timing of the analysis, or variation in tuft cell responses to different nematode species. Nevertheless, significant increases in tuft cell frequency were observed after infection with both nematodes.

The tissue sections used in IHC were taken from the abomasal fundic region where *T. circumcincta* and *H. contortus* adult parasites predominantly localize. We also examined tuft cell numbers in different abomasal regions, by scoring sections from the cardiac (anterior), fundic (mid) and pyloric (posterior) regions following *T. circumcincta* infection. The percentage of POU2F3^+^ cells was greater in all three regions post-infection relative to helminth-naïve animals (**Supplementary Figure 4**). Variation in the frequency of tuft cells in the different regions was observed between individual infected sheep, but across all animals there was no significant difference in the percentage of POU2F3^+^ cells across the different regions (P>0.05). In further studies we focused on the fundic region as representative of the general abomasal tuft cell response.

### Gene Expression Profiling of Ovine Abomasal Epithelial Cells by Single Cell RNA Sequencing (scRNA-Seq)

To establish how ovine tuft cells may be activated and respond to nematode infection, RNA profiling was carried out. As surface-expressed markers are not currently available for ovine tuft cells, fluorescent-activated cell sorting (FACS) using surface markers (5), was not possible. We therefore performed scRNA-seq to determine the gene expression profile of tuft and other epithelial and immune cells in the abomasal mucosa following nematode infection. Informed by our IHC data, tissue from the abomasal fundic region was collected at day 21 post-*T. circumcincta* infection from two sheep (**Supplementary Table 1**) and subjected to high throughput droplet-mediated scRNA-seq using the 10x Genomics Chromium platform (https://www.10xgenomics.com/products/single-cell/) (24). Cell Ranger analysis showed that approximately 9,500 cells were sequenced from each sample. Sheep sample 1 produced over 503 million reads that mapped to the ovine (92%) and bovine (72%) genomes. More reads mapped to the bovine transcriptome (43%) compared to the ovine transcriptome (33%), due to better annotation of the bovine genome. Sheep 2 sample produced over 287 million reads, of which 45% and 40% mapped to the bovine and ovine transcriptomes, respectively. Approximately 17,000 genes were detected from each sample with median unique molecular identifier (UMI) counts of approximately 4,000 per cell.

### scRNA-Seq Identifies Distinct Ovine Epithelium and Immune Cell Types

**Figure 3:**
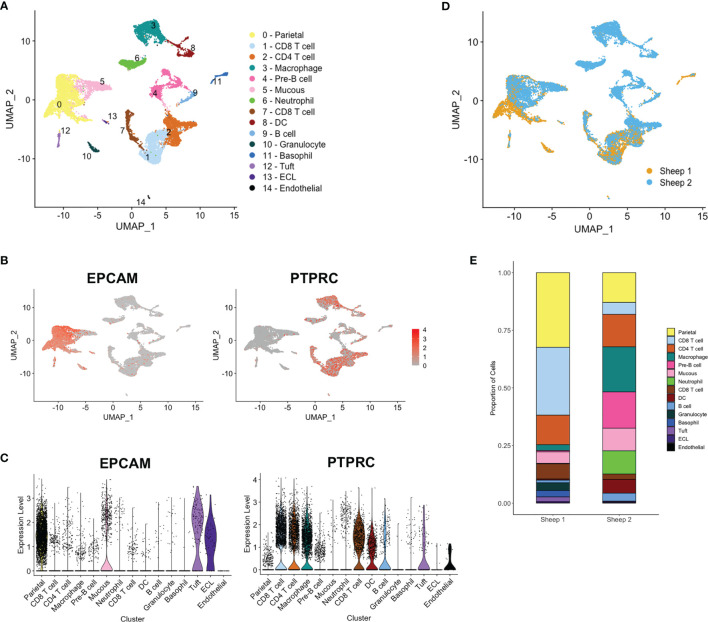
Single cell RNA sequencing (scRNA-seq) identifies distinct ovine abomasal cell clusters. **(A)** UMAP (Uniform manifold approximation and projection) plot of cell clusters identified from integrated data from sheep 1 and 2 following mapping to bovine genome. DC - dendritic cells, ECL - enterochromaffin-like cells. Cell clusters were putatively identified by expression of marker genes (see **Supplementary Table 6**). Expression of epithelial cell marker *EPCAM* and immune marker *PTPRC* (*CD45*) across all clusters, shown by **(B)** UMAP and **(C)** violin plots. **(D)** UMAP plot of sheep 1 and 2 overlapped. **(E)** Relative proportion of cells within each cluster for sheep samples 1 and 2.

Detailed analysis and clustering of scRNA-seq data was carried out using R toolkit Seurat 3.0 (http://satijalab.org/seurat/) (25). Ovine and bovine mapped data identified similar numbers of cell clusters; due to the higher number of mapped reads and better annotation, we focused on the bovine data. Datasets from the two samples were integrated using the canonical correlation analysis (CCA) method (26) and hierarchical clustering generated 15 distinct groups, (see **Figure 3A**). Based on *POU2F3* expression, a tuft cell cluster was identified and all clusters were annotated as far as possible (**Figure 3A**) based on expression of marker genes identified here (**Supplementary Table 5** **)** and in previous studies of epithelial (31), tuft (9, 32) and immune cell genes. Clusters were enriched for epithelial cell adhesin *EPCAM* or immune cell marker *PTPRC* (*CD45*) (**Figures 3B, C** **)**, except for clusters 4 and 10, identified as pre-B cells and granulocytes, respectively, in which few cells expressed either marker.

Most clusters showed overlap between samples from the two animals (**Figure 3D**) however fewer epithelial cells and more immune cell populations, particularly tissue macrophages, pre-B cells and neutrophils were contributed by sheep 2 (**Figures 3D, E** **).** At necropsy, sheep 2 showed gross inflammation of the abomasal mucosa, not observed in sheep 1, and had a higher adult worm burden and fecal egg count than sheep 1 (10,880 vs 6,910 adult worms and 432 vs 45 eggs per gram feces, for sheep 2 vs sheep 1, respectively). While these observations may explain the greater proportion of immune cell types sequenced in sheep 2, gene expression profiles were similar within the common cell clusters from the two sheep.

### A Cell Atlas for the Ovine Abomasal Mucosa

**Figure 4:**
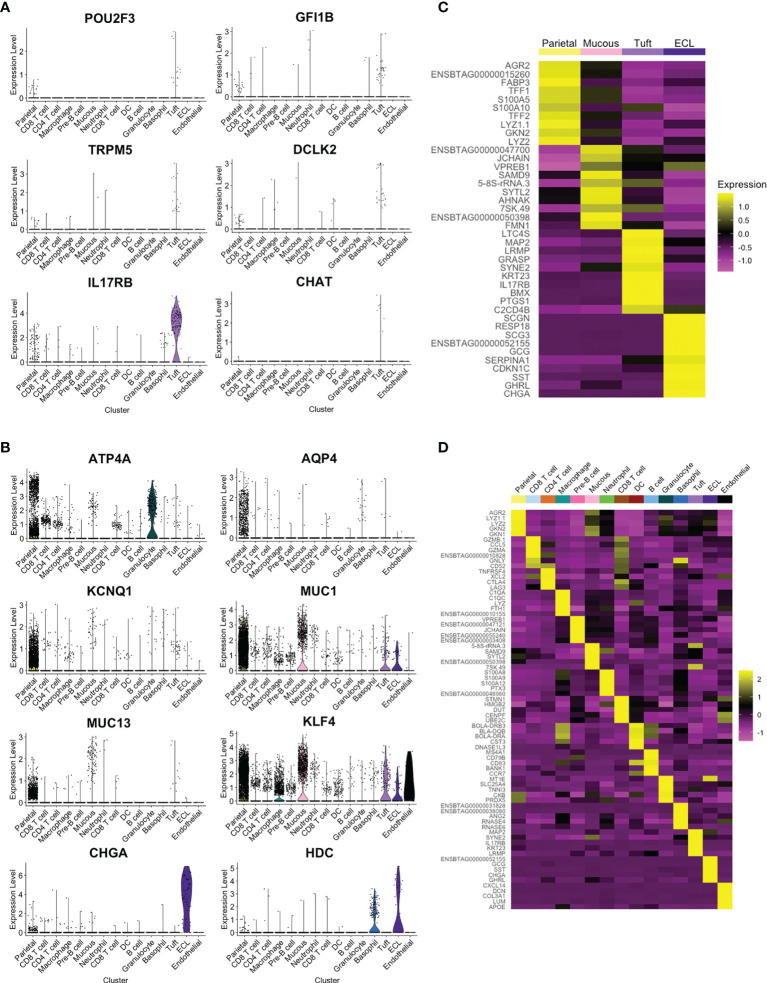
Identification of tuft cell and additional cell clusters in ovine abomasum from scRNA-seq data. **(A)** Violin plot showing log-normalised expression values of canonical tuft cell marker genes across all cell clusters. **(B)** Log-normalised expression of selected marker genes, identified previously (31), for putative parietal (*ATP4A, AQP, KCNQ1*), mucous (*MUC1, MUC13, KLF4*) and enterochromaffin-like (ECL) (*CHGA, HDC*) cells across all clusters, shown by violin plot **(C)** Heatmap of top 10 marker genes in *EPCAM-*enriched cell clusters. **(D)** Heatmap of top five marker genes in all ovine abomasal mucosa cell clusters identified (see **Supplementary Table 5** for full list of enriched genes in each cluster and **Supplementary Table 6** for selected marker gene list).

Four clusters showed strong expression of *EPCAM* and, based on expression of previously reported marker genes (9, 31, 32), were putatively identified as parietal, mucous, tuft and enterochromaffin-like (ECL) cells (**Figure 3C** and **Supplementary Table 6**). The tuft cell cluster represented 1.1% of the total cells and 3.6% of epithelial cells (**Supplementary Table 7**), and showed enriched expression of canonical tuft cell genes *POU2F3*, *GFI1b*, *TRPM5* and IL-25 receptor *IL17RB* (**Figure 4A**). *TRPM5* was expressed by a relatively low number of tuft cells (19 out of 189 tuft cells by scRNA-seq) which may, in part, explain the failure to detect TRPM5 by IHC. *POU2F3* and *GFI1B* were also not detected in all cells of the putative tuft cell cluster, which likely reflects low expression of these transcription factors. Haber et al. (32) also reported a low percentage of cells expressing some murine canonical tuft cell genes. The identity of the ovine tuft cell cluster was supported by highly enriched expression of additional tuft cell marker genes (see below).

In contrast to murine SI tuft cells (4, 32), *DCLK-1* was not identified in the ovine tuft cell cluster, despite being present in the ovine and bovine genomes. Tuft cell expression of *DCLK-2*, a paralog of *DCLK-1*, was observed (**Figure 4A**), and confirmed by *in situ* hybridisation using RNAscope (see below). Expression of *CHAT*, which encodes choline acetyltransferase and is highly enriched in murine SI tuft cells (5, 32), was almost exclusive to ovine tuft cells, but detected in only a small number (7/189) (**Figure 4A**). *IL25*, a key Th2 alarmin produced by tuft cells in mice, was not identified in the tuft cell cluster, possibly due to low levels of gene expression and/or the timing of analysis. However, as in the mouse, ovine tuft cells express high levels of the IL-25 receptor, *IL17RB* (**Figure 4A**). **Figure 4B** demonstrates enriched expression of selected marker genes from a previous study of gastric epithelial cells (31) and the top 10 marker genes of the four *EPCAM*-enriched cell clusters are shown by heat map in **Figure 4C**.

Clusters enriched for immune marker *PTPRC* (encoding CD45) could be putatively identified as T cells expressing *CD4* or *CD8*, B or pre-B cells expressing *CD19*, *CD40, CD79a/b*, *J chain* and/or *VPREB*, or myeloid cells (**Supplementary Figure 5** and **Supplementary Table 6** **)** and the top five enriched genes expressed in each cluster are shown in **Figure 4D**. The complete list of marker genes is shown in **Supplementary Table 5**, resulting in the first gene expression atlas of the ovine abomasal mucosa at the single cell level, available at http://cellatlas.mvls.gla.ac.uk/Ovine_Abomasum.

### Leukotriene and Prostaglandin Pathways Are Conserved Across Tuft Cells but G-Protein Coupled Receptors Are Divergent

**Figure 5:**
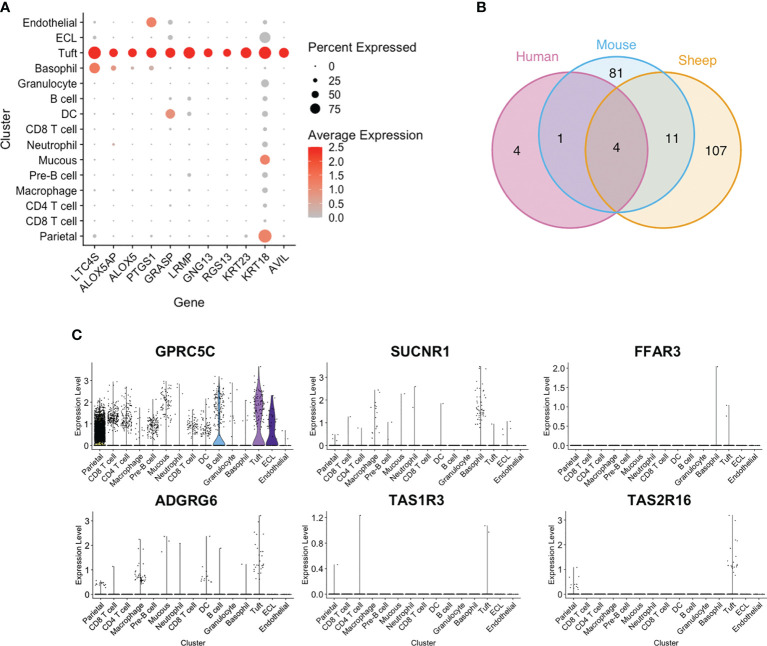
Conservation and divergence of genes associated with tuft cell function. **(A)** Dot plot showing log-normalised expression of selected tuft cell genes across clusters. **(B)** Venn diagram of ovine abomasal and mouse SI tuft cell enriched genes (Log2 fold change >0.5; FDR < 0.05), and genes conserved in human tuft cells from different tissues. Ovine, human and mouse data from this study, and (9, 32), respectively. The four genes conserved in all three species are *AVIL, ALOX, ALOX5AP, PTGS1*; the 11 genes conserved in sheep and mouse are *GNG13, LRMP, LTC4S, RGS13, KRT18, KRT23, BMX, SPIB, SH2D7, TSPAN6, TMEM45B*. **(C)** Violin plot of log-normalised expression of *GPCRs* across all clusters.

Among the top tuft cell-enriched genes were those encoding leukotriene C4 synthase (LTC4S), prostaglandin-endoperoxide synthase 1 (PTGS1), arachidonate 5-lipoxygenase (ALOX5) and arachidonate 5-lipoxygenase activating protein (ALOX5AP), all of which are involved in the synthesis of leukotriene and prostaglandin inflammatory mediators (**Figures 4C** and **5A**). In addition, advillin (*AVIL*) and keratin 23 (*KRT23*) were highly enriched in ovine tuft cells, while keratin 18 (*KRT18*) was abundant, but not specific to tuft cells (**Figure 5A**). These have been identified as markers of murine SI tuft cells (14, 32), with advillin localizing to the apical tuft structure (33). Genes encoding regulators and mediators of signal transduction were also significantly enriched in ovine tuft cells, including Grp1-associated scaffold protein GRASP, inositol 1,4,5-triphosphate receptor-associated protein LRMP, guanine nucleotide-binding subunit gamma-13 GNG13, and Regulator of G Protein Signalling RGS13 (**Figure 5A**). Importantly, these ovine abomasal tuft cell enriched genes are among the most highly expressed genes in tuft cells from mouse SI (32) and are conserved in human tuft cells from different tissues (9). **Figure 5B** shows the overlapping set of tuft cell genes from the three species, with four genes (*AVIL, ALOX, ALOX5AP, PTGS1*) conserved in all three species, and 11 genes (*GNG13, LRMP, LTC4S, RGS13, KRT18, KRT23, BMX, SPIB, SH2D7, TSPAN6, TMEM45B*) conserved in ovine and murine tuft cells. This documents the conservation of signaling and inflammatory pathways in tuft cells across species and in different tissue locations.

In contrast, we found that G-protein coupled receptors (GPCRs), which may enable tuft cells to ‘sense’ parasites, diverge between tissues and species. Murine SI tuft cells are enriched for free fatty acid receptor *FFAR3* (*GPR41*), succinate receptor *SUCNR1* (*GPR91*) and orphan receptor *GPRC5C* (32, 34). However expression of *SUCNR1* was very low in ovine abomasal tuft cells (**Figure 5C**), and was expressed mostly by cells in the basophil/mast cell cluster. *GPRC5C* was highly, but not specifically expressed in ovine tuft cells, while *FFAR3* was detected in very few abomasal cells overall (3/16,891 total cells), as was taste receptor *TAS1R3* (**Figure 5C**). This receptor is highly expressed in murine gastric tuft cells, particularly at the “gastric groove”, a tissue fold between the fundus and corpus of the rodent stomach (35), and in murine tuft cells in the distal SI (36). Notably, taste receptor *TAS2R16* and adhesion receptor *ADGRG6* (*GPR126*) were specifically enriched in ovine tuft cells (**Figure 5C**). Our scRNA-seq data are important in showing that while there is conserved expression of some *GPCRs* (*TAS2R16, GPRC5C*) in the SI and abomasum between species, others are expressed only in distinct GI locations, and suggest both tissue- and species-specific sensing. Therefore, while the downstream mediators of tuft cell function have been retained across species and GI regions, the ‘sensing’ receptors have diverged.

### Trajectory Analysis Identifies Subsets of Ovine Tuft Cells

**Figure 6:**
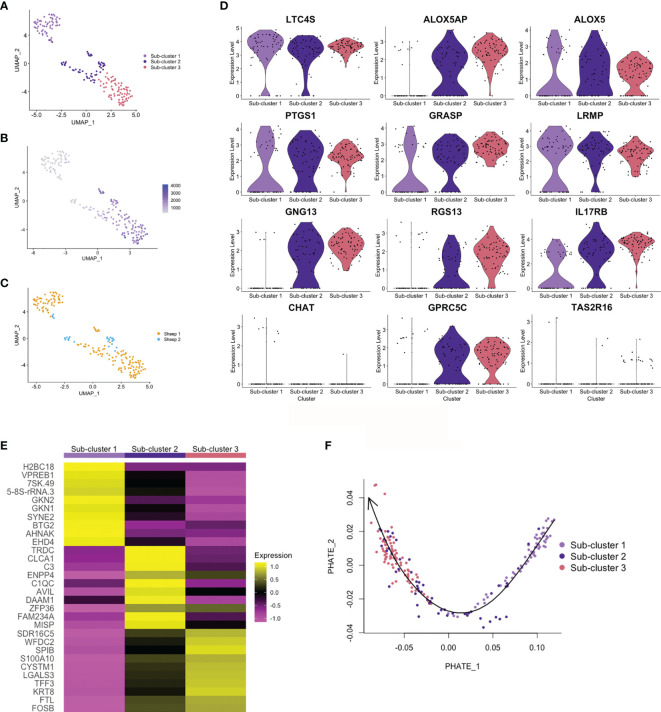
Trajectory analysis of ovine abomasal tuft cell sub-clusters. **(A)** UMAP plot of ovine tuft cell sub-clusters 1, 2, 3. **(B)** UMAP plot showing gene expression (UMI) across sub-clusters. **(C)** UMAP plot of sheep 1 and 2 tuft cell sub-clusters overlapped. **(D)** Violin plots showing log-normalised expression of selected tuft cell genes across sub-clusters. **(E)** Heatmap of top 10 marker genes in ovine tuft cell sub-clusters (see **Supplementary Table 8** for full gene lists). **(F)** PHATE (Potential of Heat-diffusion for Affinity-based Transition Embedding) plot of sub-cluster gene expression. Arrow on Slingshot line shows suggested direction of lineage, added manually, starting at sub-cluster 1.

Haber et al. (32) identified two sub-clusters of differentiated tuft cells in murine SI: tuft-1, neuronal like, and tuft-2, expressing greater levels of immune genes (*PTPRC* (*CD45*) and *TSLP*). In the airways, two tuft cell sub-clusters were also defined, based on expression of genes associated with taste transduction (tuft-1) or leukotriene biosynthesis (tuft-2) (37). From ovine abomasal tuft cell scRNA-seq data, we identified three sub-clusters (**Figure 6A**), but these were not defined on expression of neuronal or immune-related genes. Sub-clusters 2 and 3 showed greater levels of overall gene expression (higher UMI) compared to sub-cluster 1 (**Figure 6B**). Most tuft cells from sheep 2 were in sub-cluster 2 (**Figure 6C**); whether sub-cluster type may influence infection outcome will require analysis from additional animals. All three sub-clusters were enriched for canonical tuft cell genes, however sub-cluster 1 showed lower expression of *ALOX5AP*, G-protein signaling genes *GNG13* and *RGS13*, and *GPCRs* than sub-clusters 2 and 3, but greater expression of *CHAT* (**Figure 6D**). The top 10 marker genes of the three sub-clusters are shown **Figure 6E** and all sub-cluster marker genes are listed in **Supplementary Table 8**. The top genes in sub-cluster 1 are involved in regulation of gene expression and cell proliferation (*7SK.RNA, BTG2, AHNAK, EHD4*), while those of sub-clusters 2 and 3 are associated with cell activation, proliferation, cellular interaction and immunity (*CLCA1, C3, ENPP4, DAAM1, SPIB, S100A10, LGALS3, TFF3*).

Trajectory inference and pseudotime analysis identified an expression trajectory, which we propose may start from sub-cluster 1 (**Figure 6F**), and gene expression across pseudotime showed dynamic patterns of up or down regulation (**Supplementary Figure 6**). Gene Ontology (GO) analysis identified enrichment of genes associated with regulation of cell proliferation, extracellular space, cell surface and extracellular exosome in sub-cluster 1 (False Discovery Rate (FDR) <0.05)(e.g. *GKN1, GKN2, AHNAK, ABL2, TSC22D2, NFKB1A*), while GO term extracellular exosome (e.g. *S100A10, LGALS3, TSPAN1, TFF3*) was enriched for sub-cluster 3 genes. From this trajectory analysis, albeit from a low number of cells, we suggest that sub-clusters 1, 2 and 3 may represent early, intermediate and mature tuft cells, respectively.

### RNAscope Confirms Expression of Ovine Tuft Cell Genes

**Figure 7:**
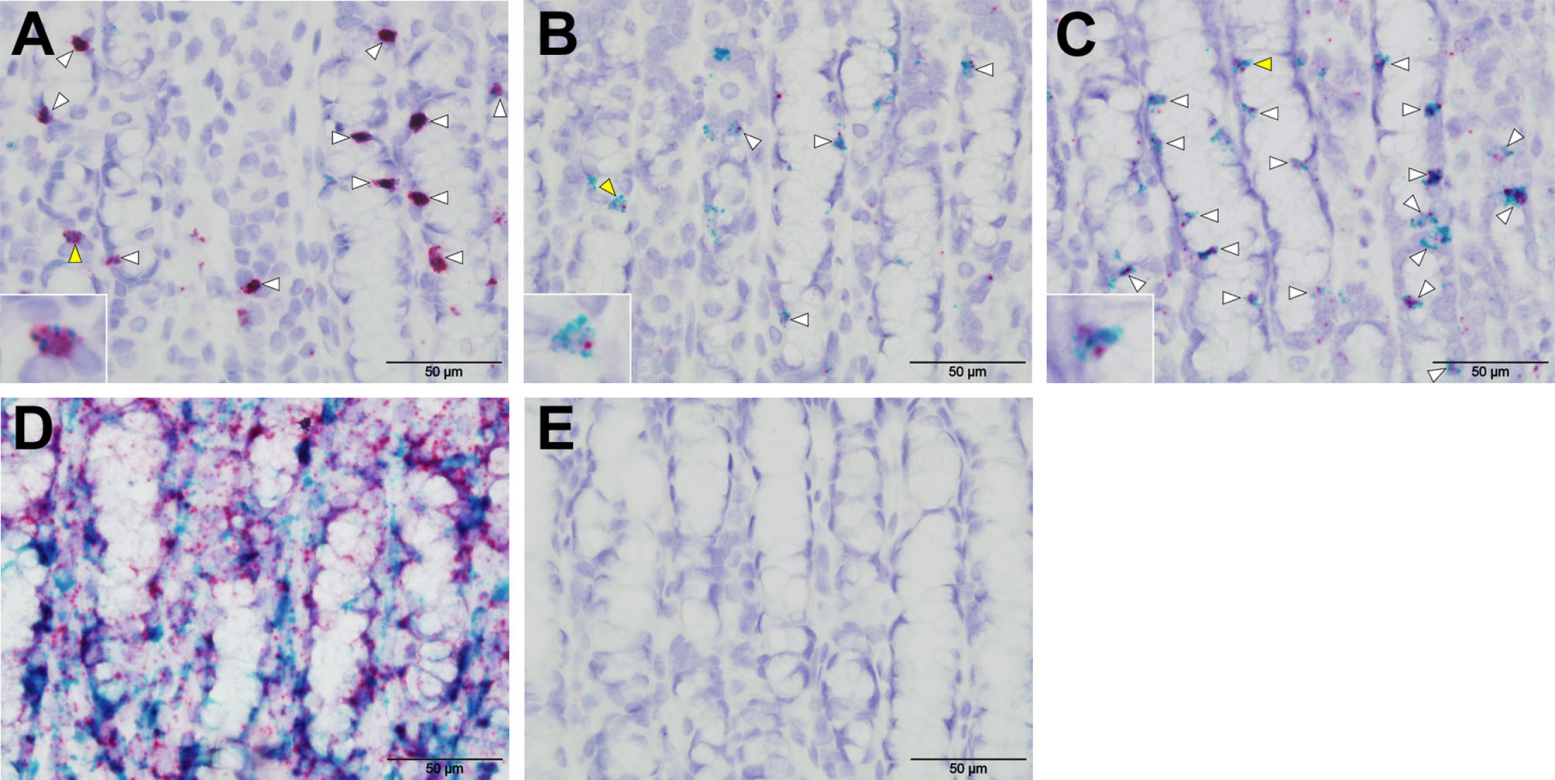
RNAscope validates scRNA-seq of ovine tuft cell gene expression. Co-expression of probes to *POU2F3* (green) and **(A)** *IL-17RB* **(B)** *TASR16* **(C)** *DCLK-2* (all red) in ovine abomasal tissue following *H contortus* infection. Arrowheads show probe co-localization, with yellow arrows indicating the cells represented in the insets. **(D)** Signal from positive control probes to ovine beta actin (*actb*, green) and ovine peptidylprolyl isomerase (*ppib*, red). **(E)** No labelling with negative control probe *DapB* from *Bacillus subtilis*.

RNAscope *in situ* hybridisation was carried out to confirm expression of selected tuft cell genes identified by scRNA-seq. Tissue fixation, probe hybridisation and signal detection were optimized using positive control probes to ovine beta actin (*actb*) and ovine peptidylprolyl isomerase (*ppib*) **Figure 7D**), while negative control probe (*DapB*) showed no hybridisation, as expected (**Figure 7E**). Probes specific to *POU2F3* localized to numerous cells in ovine abomasal tissue sampled following *H. contortus* infection, and co-localized with probes to *IL17RB, TAS2R16* and *DCLK-2* (**Figures 7A–C**). This supported co-expression of tuft cell genes identified by scRNA-seq. The signal was more abundant for *IL17RB*, than for *DCLK-1* and *TAS2R16*, consistent with scRNA-seq data. To confirm expression and co-localization of tuft cell genes in additional samples, we also tested the probes on available tissue from naive sheep. Similar to the tissue sampled post-helminth infection, probes to *IL17RB, TAS2R16* and *DCLK-2* co-localized with *POU2F3* probe, but in a lower number of cells, as expected from our IHC data (**Supplementary Figure 7**). Most tuft cells were labelled with co-localized probes, while some singly labelled cells were also observed, suggesting possible heterogeneity between tuft cells, expression in additional cell types and/or differences in orientation of the cells during sectioning. RNAscope validated the scRNA-seq data and confirmed expression and co-localization of tuft cell genes.

## Discussion

Here we identify the presence, expansion and maturation of tuft cells in the ruminant abomasum following infection with two important parasitic nematodes. Abomasal epithelial tuft cells were identified by co-expression of POU2F3 and GFI1b, and scRNA-seq detailed the gene expression profile of these and other cell populations within the abomasal mucosa. This established a gene expression atlas searchable at the single cell level as a resource for further defining abomasal responses. Our data support a role for tuft cells in mucosal immunity in the ruminant stomach, and indicate that while the downstream mediators of tuft cell function are conserved across species and tissues, a different repertoire of receptors is involved in parasite sensing. These findings will help inform development of prophylactic treatment to control these important infections, and have relevance to other host species. Furthermore, observed tuft cell heterogeneity suggests potential functional differences in these cells, which warrants further investigation under steady state and following parasite infection.

The increase in tuft cell numbers following *T. circumcincta* infection in sheep is similar to that following *N. brasiliensis* infection in mice (4). An increase was first observed when immature adult worms are emerging from the gastric glands (day 10 p.i.), suggesting that the response requires parasites to be in close proximity to the luminal surface and/or is associated with tissue damage as parasites emerge. An important role for ovine tuft cells in stimulating a type-2 response is supported by our previous work comparing two breeds of sheep highly resistant or susceptible to *H. contortus* infection. Animals of the resistant breed had significantly greater numbers of POU2F3^+^ cells and Th2 cells in the abomasal epithelium compared to susceptible animals following infection (21) and a strong correlation between ovine POU2F3^+^ cells, Th2 frequency and nematode clearance was identified.

Amongst the most highly expressed tuft cell genes in sheep (this study) and mice (32, 34) were those encoding enzymes for leukotriene and prostaglandin synthesis and intracellular signaling, suggesting that tuft cells in different species and GI regions use the same mediators to initiate a type-2 response. McGinty et al. (38) demonstrated synergy between cysteinyl leukotrienes (cysLT) and IL-25 in stimulating ILC2, with both mediators dependent on TRPM5. The rapid synthesis of cysLTs and their shorter half-life compared to IL-25 may enable cysLTs to act as a rapid on/off switch (38) and may also explain their detection by scRNA-seq.

While mediators of tuft cell activity are conserved, the receptors that sense stimuli seem to be divergent. We found no or very low expression of *SUCNR1, FFAR3* and *TAS1R3*, receptors previously identified in murine SI tuft cells (32, 34, 36). *SUCNR1* is essential for tuft cell expansion following succinate treatment or infection with the protist *Tritrichomonas* (34, 39, 40). However *SUCNR1* knockout mice can still expel *N. brasiliensis*, which primarily infects the proximal SI. It was suggested that SUCNR1 may not be required or is redundant for sensing SI nematode infection (34, 39) and our data indicate that SUCNR1 is not involved in tuft cell sensing in the abomasum. Similarly, we detected very low expression of sweet/umami receptor *TAS1R3* in ovine abomasal tuft cells, in contrast to its high expression in murine stomach and distal SI cells (35, 36).

Taste receptor *TAS2R16*, adhesion receptor *ADGRG6* (*GPR126*) and orphan receptor *GPRC5C* were enriched in abomasal tuft cells, and our data suggest these may be the main receptors involved in sensing nematode infection in the abomasum. *TAS2R16* from human and ovine/bovine is homologous to murine *TAS2R143*, which was among a small number of *TAS2R* genes upregulated in the SI following infection with the nematode *Trichinella spiralis* or after treatment of intestinal organoids with IL-13 (41). Expression of *TAS2* receptors was not detected in naive mice (34, 36) nor following infection with the SI nematode *H. polygyrus* (32), suggesting possible induction in response to specific infection. ADGRG6 is proposed to act as a mechanosensory receptor (42). The enriched expression of this receptor on ovine tuft cells following infection suggests that the anti-helminth tuft cell response may involve both chemical and mechanical signal transduction. The ovine tuft cell receptors identified here are being expressed in HEK cells to test activation by nematode excretory-secretory (ES) products (Gillan et al., unpublished). This will help determine ligands involved in sensing GI nematodes in the abomasum that may be exploited to potentiate type-2 responses against these important parasites.

Surprisingly, only a few abomasal tuft cells expressing *CHAT*, required for acetylcholine (ACh) synthesis, were identified by scRNA-seq. CHAT^+^ tuft cells were previously reported in mouse stomach (43) and SI (6, 32) and in human SI and pancreatic-biliary tract, but not in human stomach cells (9, 44). Enrichment of tuft cells co-expressing CHAT and DCLK-1 occurs in the “gastric groove”, a tissue fold between the fundus and corpus of the rodent stomach (43, 45). This groove is not present in the ruminant abomasum nor human stomach, and may explain the paucity of *CHAT*-expressing tuft cells in the fundic region of the ovine abomasum observed here.

ACh can regulate changes in smooth muscle activity in response to inflammation or bitter substances, mediating protective reflex responses (46, 47). Recent studies also demonstrated a functional role for ACh from ILC2 in promoting type-2 responses during nematode infection (48, 49). Whether ACh produced by tuft cells may be involved in smooth muscle activity and/or induction of immunity is currently unknown. Interestingly, parasitic stages of GI nematodes secrete acetylcholinesterase (AChE), which is thought to play a role in immunomodulation (50, 51). AChE activity was notably higher in parasitic nematodes residing in the SI, compared to *H. contortus* and *T. circumcincta*, which are abomasal dwelling parasites (50, 52). It is possible that SI nematodes may have a greater requirement to adapt to host ACh activity than abomasal/stomach dwelling species and future studies will compare *CHAT* expression in ovine SI and abomasal tuft cells. To date, purification of ovine tuft cells has not been possible due to lack of information on surface markers. Our scRNA-seq data has helped identify putative surface markers, such as IL-17RB and LRMP, to which antibodies can be generated to enable detailed comparison of tuft cells across tissues and at different time-points post-infection.

Trajectory analysis of scRNA-seq data identified three tuft cell sub-clusters which we propose may represent different stages of maturation. The putative “early” sub-cluster was enriched for genes involved in regulating cell proliferation, *via* cell cycle repression. Interestingly, these included proposed tumour suppressor AHNAK, which is potentiated by TGF-β (53), homologues of which are produced by many nematode species (54). This may represent a novel mechanism by which nematode products modulate host cell development and/or function and may extend to other epithelial cells that also express *AHNAK.* The putative “mature” sub-cluster showed greater expression of genes associated with extracellular exosomes as well as innate immunity and cell proliferation/differentiation. Our data suggest functional differences between the sub-cluster types identified here; future work will examine whether distribution of these sub-types may impact parasite clearance and repair of mucosal tissue to influence infection outcome, with relevance to livestock and human infection.

## Data Availability Statement

The datasets presented in this study can be found in **Supplementary Material** or in online repositories. scRNA-seq data are available in the ArrayExpress database at EMBL-EBI (www.ebi.ac.uk/arrayexpress), accession number E-MTAB-10231. Scripts for scRNA-seq analysis are available on request. A searchable cell atlas of scRNA-seq cluster expression data can be queried at: http://cellatlas.mvls.gla.ac.uk/Ovine_Abomasum.

## Ethics Statement

The animal study was reviewed and approved by Moredun Research Institute (UK) Animal Experiments Committee.

## Author Contributions

CB, ED, TM, RM, and PJ conceived the ideas and designed the study. KH and VG performed laboratory work. TO provided software and computational expertise. All authors analyzed data and/or provided samples and reagents. CB and KH wrote the paper with contributions from TM, ED, RM, TO, and the manuscript was reviewed and commented on by all authors. All authors contributed to the article and approved the submitted version.

## Funding

KH is supported by an Industrial Partnership PhD studentship funded by University of Glasgow, Moredun Foundation and Pentlands Science Park, UK. TM is supported by the Scottish Government’s Rural Affairs, Food and the Environment (RAFE) Strategic Research Portfolio 2016-2021. VG and SL are funded by a Wellcome Trust Collaborative Award (Ref 211814) to CB, ED, TM, PJ, and RM; SL and RM also received Wellcome Trust support through an Investigator Grant (Ref 219530), and the Wellcome Trust core-funded Wellcome Centre for Integrative Parasitology (Ref 104111). TDO is supported by Wellcome Trust grant 104111/Z/14/ZR.

## Conflict of Interest

The authors declare that the research was conducted in the absence of any commercial or financial relationships that could be construed as a potential conflict of interest.

## Publisher’s Note

All claims expressed in this article are solely those of the authors and do not necessarily represent those of their affiliated organizations, or those of the publisher, the editors and the reviewers. Any product that may be evaluated in this article, or claim that may be made by its manufacturer, is not guaranteed or endorsed by the publisher.
