# Supplementary material for: Tuft Cells Increase Following Ovine Intestinal Parasite Infections and Define Evolutionarily Conserved and Divergent Responses
Source: Front Immunol. 2021 Nov 22;12:781108. doi: 10.3389/fimmu.2021.781108 (PMC8646091; doi:10.3389/fimmu.2021.781108)
Supplement: Supplementary file 1 [file DataSheet_1.pdf]

Supplementary Table 1. Summary of sheep infection experiments

Experiment	Infection	No. of sheep sampled	Timepoint of tissue collection
IHC	Oral bolus of 50,000 L3 of *T. circumcincta*	18 infected<br>6 naïve<br>Suffolk-cross<br>12 months old male	5, 10, 21 dpi
IHC	Oral bolus of 10,000 L3 of *H. contortus*	6 infected<br>3 naïve<br>Suffolk-cross<br>11 months old male	55 dpi
scRNA-seq	Oral bolus of 50,000 L3 of *T. circumcincta*	2 infected<br>Texel-cross<br>6 month old male	21 dpi

IHC = immunohistochemistry; dpi = day post-infection

Supplementary Table 2. Details of antibodies used for immunohistochemistry

Target	Host and antibody type	Source	Dilution
POU2F3	Rabbit pAb	Sigma-Aldrich, HPA019652	1:200 IF<br>1:500 IHC
GFI1B	Mouse mAb	Santa Cruz Biotechnology, Clone B-7, Sc-288356	1:100
DCLK-1	Rabbit pAb	Abcam, ab31704	1:50 – 1:2000
TRPM5	Rabbit pAb	Aviva Systems Biology, ARP35242 _P050	1:200
Villin	Mouse mAb	Chemicon International, MAB1671	1:200
IgG pAb control	Rabbit pAb	Sigma-Aldrich, I5006	Matched to conc. of 1y Ab
$IgG2_b$ mAb control	Mouse mAb	Moredun Research Institute, Clone AV29	1:100
Mouse IgG_1	Monoclonal	VPM21 (IgG_1), Institute of Animal Health, Compton, UK	1:100
Secondary mouse-488	Polyclonal	Abcam, Ab150117	1:500
Secondary rabbit-546	Polyclonal	Thermofisher Scientific, A11010	1:500
Alexa Fluor™ 488 Tyramide SuperBoost™ Kit	N/A	ThermoFisher Scientific, B40912	N/A

Supplementary Table 3. Conservation of tuft cell marker proteins in human, mouse and sheep

Protein	Function	Human	Mouse	Sheep	% ID to Sheep (Human, Mouse)
POU2F3	Transcription Factor	EAW67503	XP_011240722	XP_014956448	94, 92
GFI1B	Transcription Factor	NP_001364233	NP_031240	XP_014949605	88, 84
DCLK-1	Kinase	NP_001182345	NP_01104521	XP_012039965	99, 98
TRPM5	Cation channel	EAX02514	XP_017177873	XP_027815546	83, 80

Supplementary Table 4. Percentages of POU2F3+ cells in total epithelium cells in naïve sheep (day 0) and at days 5, 10 and 21 following *T. circumcincta* infection.

dpi	Animal number	Tuft cells as a % of epithelial cells in each image (x40 magnification) Area 1	Area 2	Area 3	Area 4	Area 5	Mean % of 5 Areas	Mean %	SD	Median %
0	U1P1	2.83	0	0.43	3.33	0.89	1.5	0.90	0.35	1.09
	U2P1	1.8	0	0	0.38	0.46	0.5			
	U3P1	0	1.8	0	1.25	0.37	0.7			
	U4P1	0.84	1.23	0.87	0.9	1.46	1.06			
	U5P1	1.55	0	0	1.4	2.62	1.11			
	U6P1	0.38	1.22	2.05	1.59	0.48	1.14			
5	136B	0.46	1.42	1.83	0.99	5.4	2.02	1.46	0.37	1.39
	034B	1.55	0.43	3.53	1.36	0.53	1.48			
	051B	0.62	2.58	1.04	1.15	1.11	1.3			
	084B	0.98	0.57	2.91	2.5	1.78	1.75			
	076B	2.73	1.94	0	0.57	0	1.04			
	107B	0.87	0.97	1.42	1.02	1.65	1.19			
10	019B	2.58	2.38	3.04	4.61	4.86	3.49	4.71	2.10	4.20
	004B	6.45	2.497	3.83	5.14	4.88	4.56			
	021B	0.43	0	3.14	4.03	11.64	3.85			
	090B	4.74	4.17	5.82	4.91	5.50	5.03			
	073B	1.78	6.75	0	2.88	1.89	2.66			
	067B	8.16	4.46	12.30	8.33	10.04	8.66			
21	103B	4.33	7.11	2.51	6.39	1.58	4.38	7.08	3.43	6.20
	072B	8.04	4.47	6.51	6.53	6.51	6.41			
	092B	14.92	8.26	6.25	7.06	14.24	10.14			
	060B	4.23	7.61	3.32	7.20	7.60	5.99			
	105B	4.37	0.52	3.68	5.11	2.92	3.32			
	049B	12.45	20.43	7.43	12.13	8.63	12.21			

dpi = day post-infection; SD = standard deviation of mean

Supplementary Table 6. Marker genes used to identify ovine abomasum cell clusters, based on enriched expression across clusters (Supplementary Table 5) and published data (9,31,32)

Cluster	Putative cell type	Marker Genes
0	Parietal	ATP4, AQP4, KCNQ1, AGR2, LYZ1.1, LYZ2, TFF1, TFF2, S100A5
1	CD8 T cell	CD3E, CD7, CD8A, CCL4, CCL5, GZMA, GZMB.1
2	CD4 T cell	CD4, CTLA4, CD52, CXCR4, IL2RA, LAG3
3	Macrophage	CD68, CD74, CXCL3, C1QC, C1QA, CTSZ, CTSS, CTSC, BOLA-DRA
4	Pre-B cell	Jchain, VPREB1, VPREB3, MZB1
5	Mucous	MUC1, MUC20, MUC13, SYTL2, KLF4
6	Neutrophil	S100A12, S100A8, S100A9, CSF3R, GPR84, NCF1
7	CD8 T cell	CD3E, CD8, STMN1
8	DC	BOLA-DRA/DQB, CD74, CD83, CXCL9, CST3
9	B cell	CD83, CD79B, CD19, CD74, CD40, CXCR4, CXCR5, MS4A1 (CD20), BANK1
10	Granulocyte	CD24, ATP4, ATP5, IL20RA
11	Basophil	CCL3, CCL2, GATA2, TPSB2, IL4, FcER1A, ANG2
12	Tuft	IL17RB, KRT23, LTC4S, PTGS1, ALOX5, LRMP, GRASP, AVIL, CD24
13	ECL	CHGA, HDC, SST, GHRL
14	Endothelial	C3, CD99, DCN

Supplementary Table 7. Number and percentage of cells in each cluster from integrated scRNA-seq data

Cluster	Cell count	Percentage of total count
Parietal	3730	22.08
CD8 T cell	2809	16.63
CD4 T cell	2277	13.48
Macrophage	1950	11.54
Pre-B cell	1452	8.60
Mucous	1271	7.52
Neutrophil	914	5.41
CD8 T cell	741	4.39
DC	572	3.39
B cell	394	2.33
Granulocyte	267	1.58
Basophil	246	1.46
Tuft	189	1.12
ECL	52	0.31
Endothelial	27	0.16

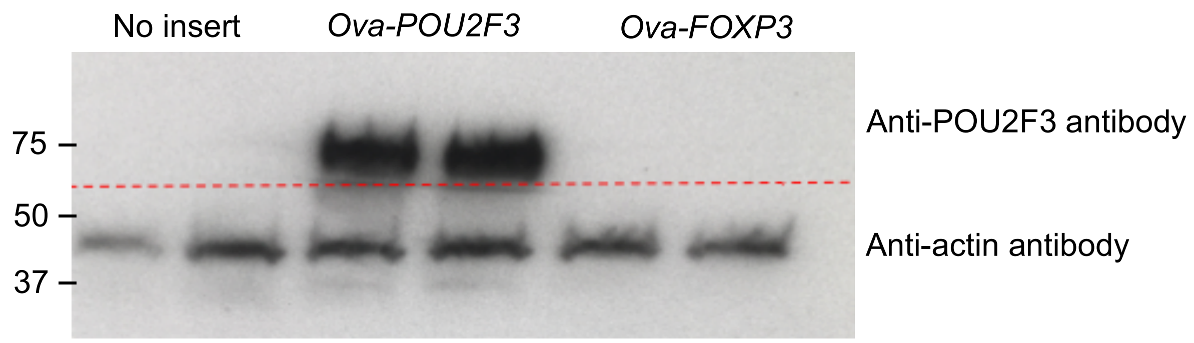

Supplementary Figure 1. Western blot showing specificity of anti-POU2F3 antibody to ovine POU2F3 protein. Cell lysates from HEK cells transfected with pCI-neo vector containing no insert sequence, *Ova-POU2F3,* or unrelated *Ova-FOXP3* gene were probed with anti-POU2F3 or anti-actin antibody and signal detected using chemiluminescent substrate. Duplicate samples were probed for each lysate.

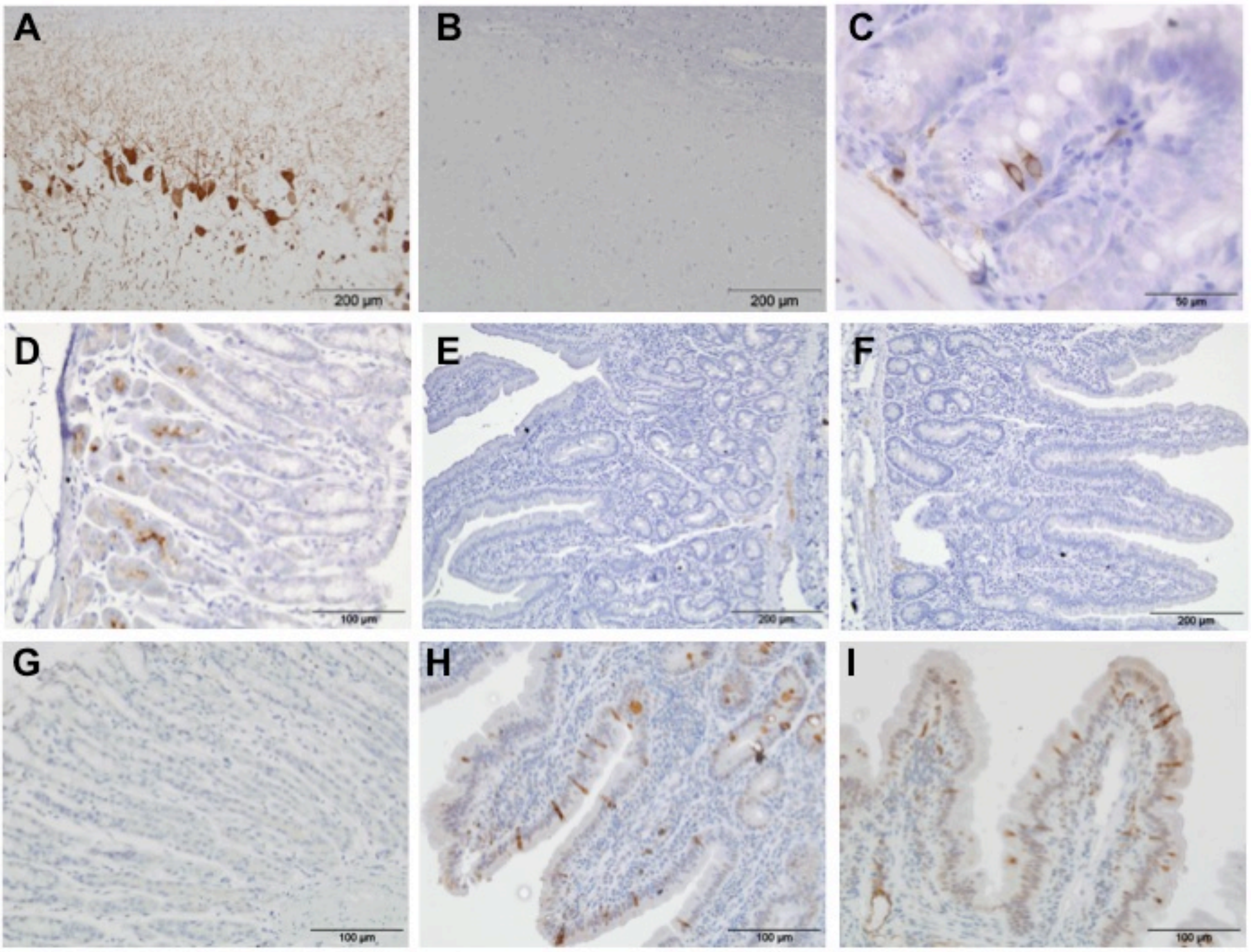

Supplementary Figure 2. DCLK-1 and TRPM-5 antibody labelling. (A) DCLK-1^{+} cells (brown) were identified in ovine neuronal cells while **(B)** IgG isotype control was negative. DCLK-1 antibody identified putative tuft cells in murine SI tissue **(C)** while non-specific or no labelling was observed in ovine abomasum **(D)**, duodenum **(E)** and jejunum **(F)**. TRPM-5 antibody does not show labelling in ovine abomasum tissue **(G)**, but localizes to putative tuft cells in the ovine duodenum **(H)** and jejunum **(I)**.

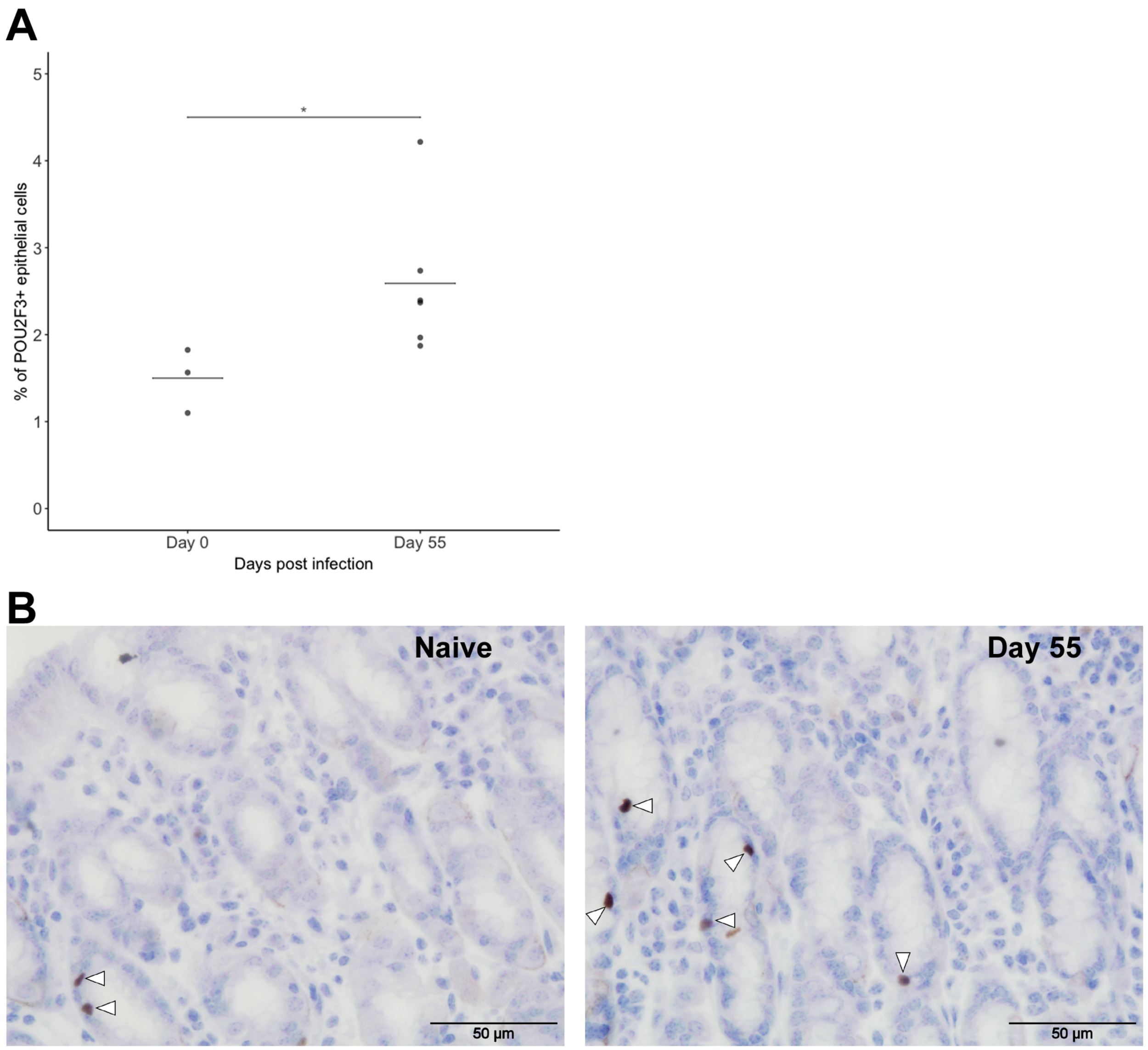

Supplementary Figure 3. Increase in abomasal tuft cell following *Haemonchus contortus* infection. (A) Percentage of POU2F3^{+} cells in abomasal epithelium in naïve sheep and at day 55 following infection with *H. contortus*. The percentage from five tissue sections was calculated from three naive (day 0) and six infected (day 55) animals ($P < 0.05$ using a two-sample t-test) Horizontal lines indicate mean value. **(B)** Representative images of POU2F3^{+} cells (brown) in the ovine abomasum epithelium from naïve sheep and at day 55 after *H. contortus* infection. White arrowheads indicate POU2F3^{+} cells.

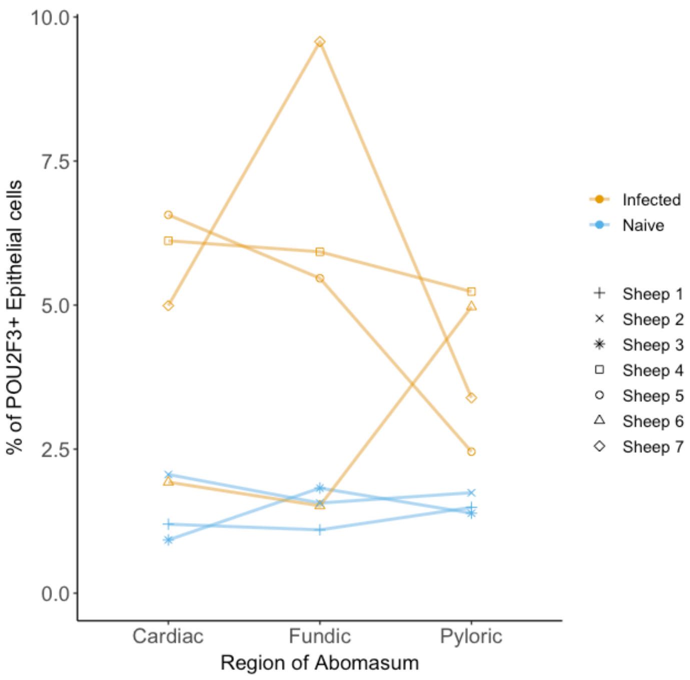

Supplementary Figure 4. Presence of tuft cells in different regions of ovine abomasum (cardiac, fundic, pyloric). Percentage of POU2F3$^+$ cells detected by immunohistochemistry across the three regions in naive sheep (n=3) and following infection with *Teladorsagia circumcincta* (n=4).

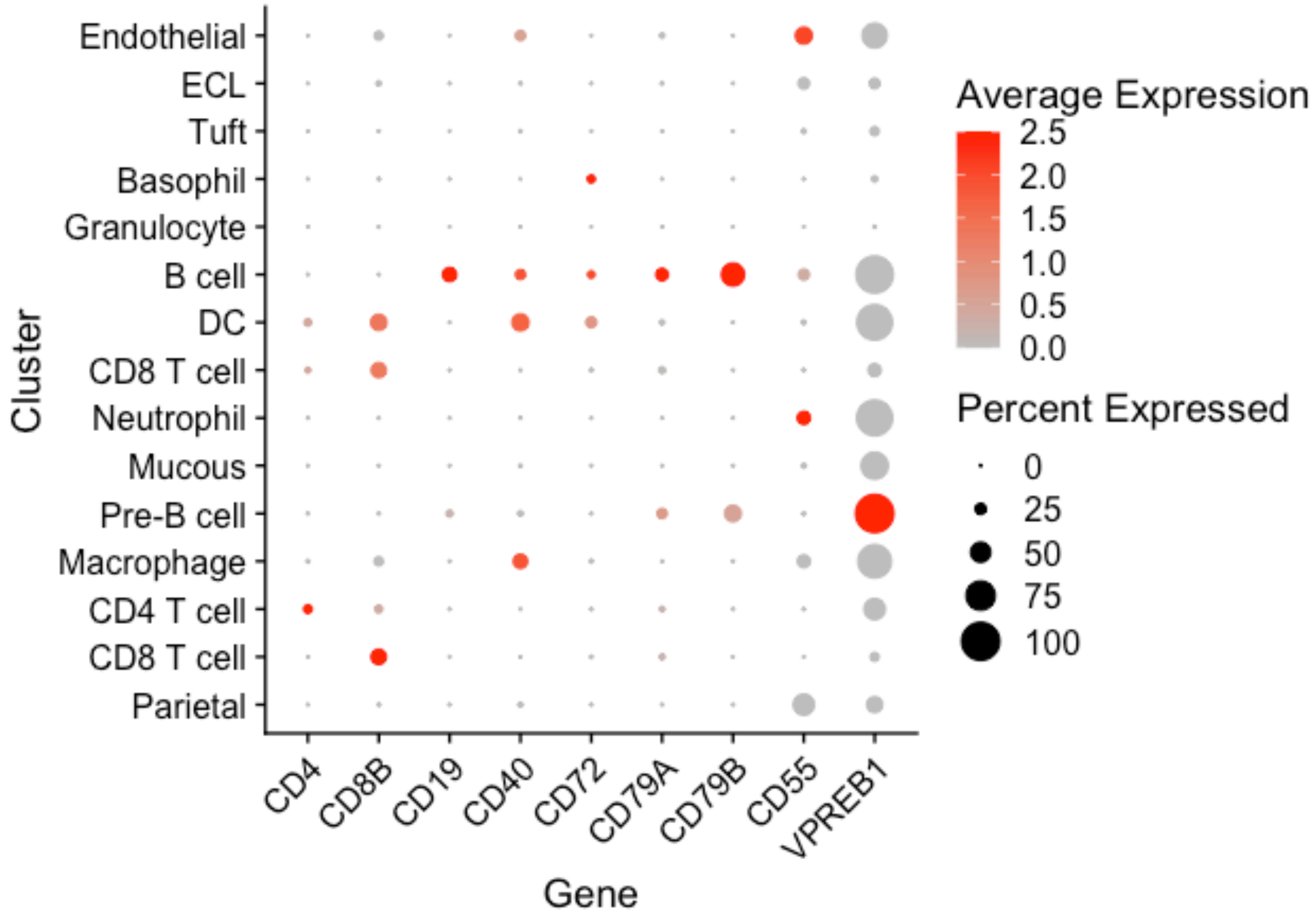

Supplementary Figure 5. Dot plot showing log-normalised expression values of immune cell marker genes across all cell clusters. Marker gene lists for each cell cluster are available in Supplementary Table 5.

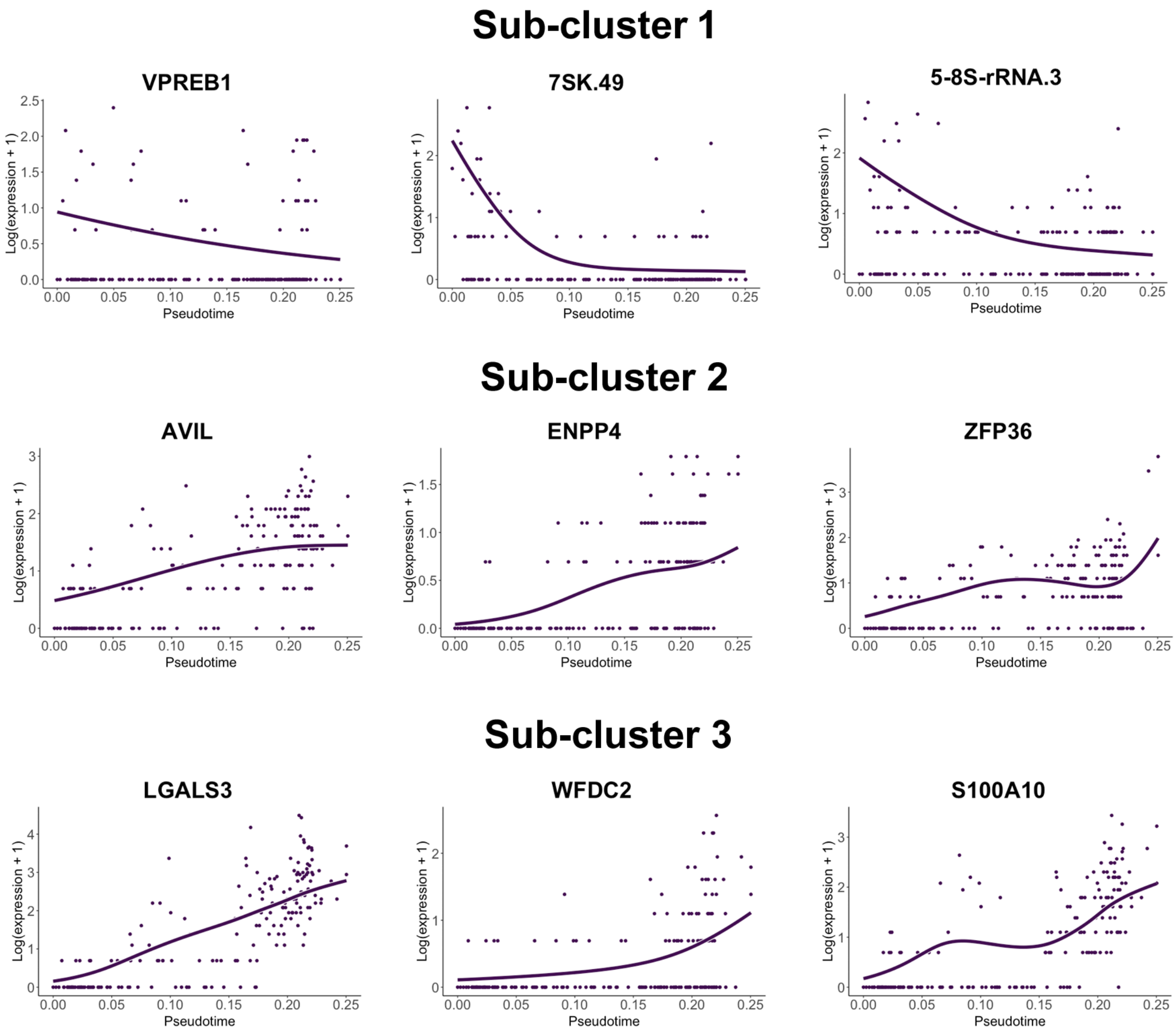

Supplementary Figure 6. Gene expression across pseudotime for three selected genes upregulated in each of the three tuft cell sub-clusters. Marker gene lists for tuft cell sub-clusters 1, 2 and 3 are available in Supplementary Table 8.

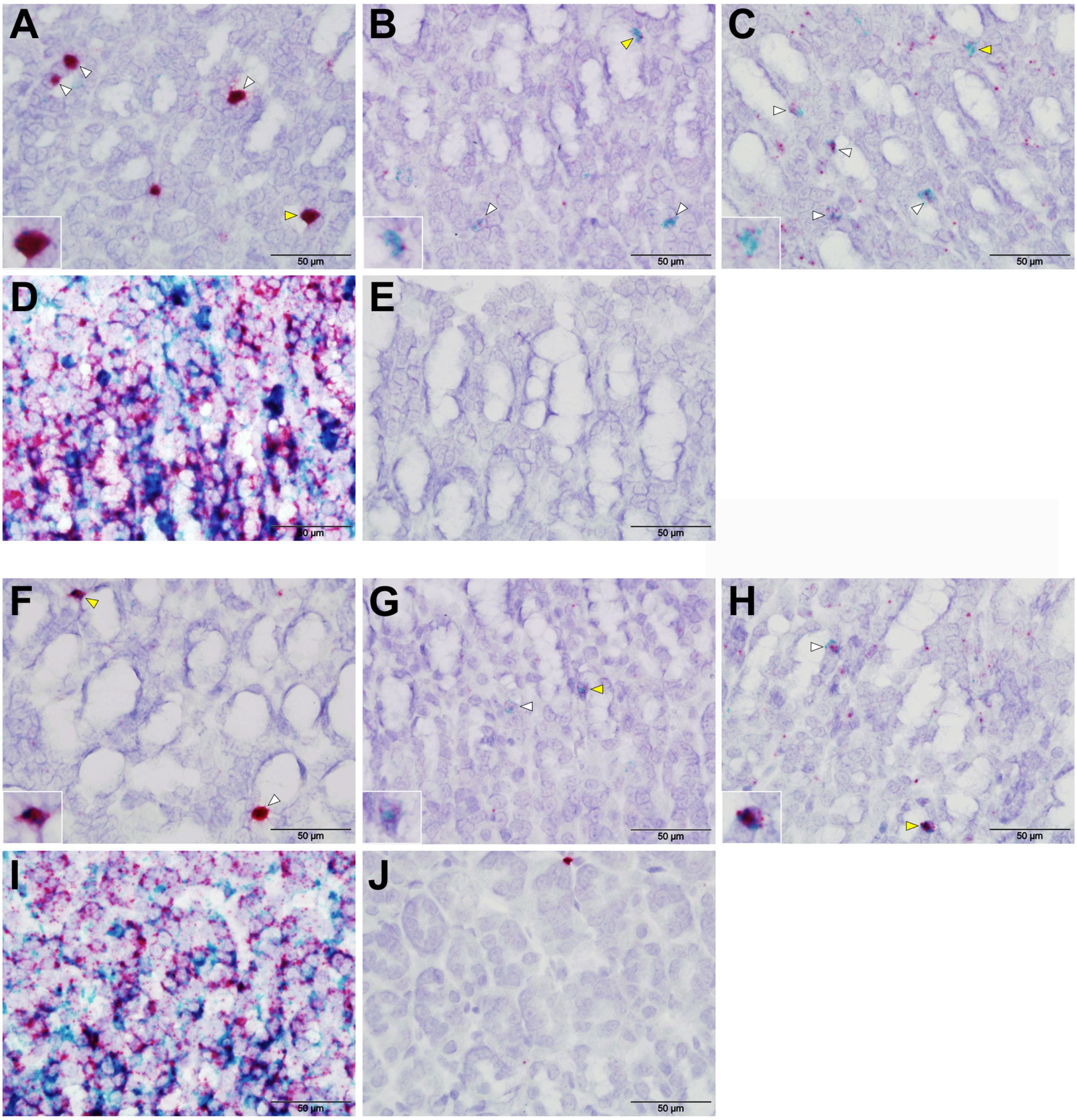

Supplementary Figure 7. RNAscope of ovine tuft cell gene expression. Co-expression of probes to *POU2F3* (green) and (**A, F**) *IL-17RB* (**B, G**) *TAS2R16* (**C, H**) *DCLK-2* (all red) in ovine abomasal tissue from two control (non-infected) sheep (numbers 12633 and 12660). This shows fewer tuft cells, but similar co-localisation of *POU2F3* with other tuft-cell genes as seen in abomasal tissue from *H. contortus* infected sheep (Figure 7). Arrowheads show probe co-localisation, with yellow arrows indicating the cells represented in the insets. (**D, I**) Signal from positive control probes to ovine beta actin (*actb*, green) and ovine peptidylprolyl isomerase (*ppib*, red). (**E, J**) No labelling with negative control probe *DapB* from *Bacillus subtilis*. Panels (**A-F**) represent tissue from sheep 12633; panels (**G-J**) represent tissue from sheep 12660.
